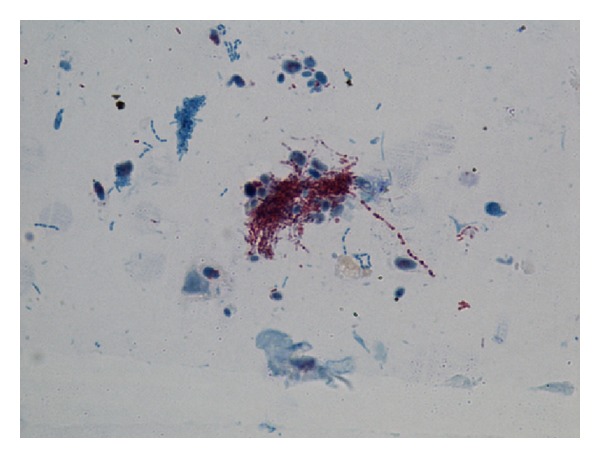# Allergic Broncho Pulmonary Aspergillosis Complicated by Nocardiosis

**DOI:** 10.1155/2012/758630

**Published:** 2012-12-25

**Authors:** Brijesh Sharma, Gopal Ghosh, Ulka Kamble, Karan Chaudhary, Ajay Chauhan, B. M. S. Lamba, Anuradha Chowdhary, B. B. Gupta

**Affiliations:** ^1^Department of Medicine, PGIMER & Dr. Ram Manohar Lohia Hospital, New Delhi 110001, India; ^2^Department of Medical Mycology, V.P. Chest Institute, University of Delhi, Delhi 110007, India

## Abstract

We describe a 70-year-old male with a history of diabetes mellitus, hypertension, and asthma who presented with increasing breathlessness for 5 months. He was diagnosed to have allergic bronchopulmonary aspergillosis (ABPA) by serological and radiographic criteria. He was treated with steroids and itraconazole. After initial improvement, he developed fever with cough and mucopurulent sputum. X-ray chest revealed multiple cavities with air fluid level. Patient was treated with antibiotics without any response. Sputum was negative for acid fast bacilli (AFB). Sputum culture for bacteria and fungus did not reveal any significant growth; however a delayed growth of Nocardia was noted on fungal plates. Modified Ziehl Nelsen stain was positive for AFB. Patient was treated with cotrimoxazole. We discuss the serological and radiological criteria of ABPA, presentation and treatment of *nocardia* pulmonary infection and other possible causes of necrotizing pneumonia in immunocompromised settings.

## 1. Case Discussion

A 70-year-old male with a history of bronchial asthma for 25 years, type 2 diabetes, and hypertension for 10 years presented with a 5-month history of increasing breathlessness. He had seen multiple physicians over this period and had been treated with various antibiotics, ciprofloxacin, levofloxacin, and, most recently amoxicillin/clavulanic acid and inhaled and oral bronchodilators with little to no improvement. At the time of admission, he complained of increasing shortness of breath for last 5 days, fever with chills for 3 days, minimally productive cough, and generalized fatigue. Over the years he had been treated with inhaled formoterol and fluticasone for asthma. He denied chronic systemic corticosteroid use. His diabetes was not controlled, though he was taking oral hypoglycaemic agents (metformin and glimepiride). His hypertension was under control on amlodipine. He had no known drug allergies and denied any family history of lung disease or allergies. There was no history of exposure to animals, environmental irritants, or tuberculosis. He denied high risk sexual behaviour and tobacco, alcohol, or intravenous drug use.

On physical examination, he was febrile with temperature of 100.4°F, pulse rate of 94/minutes, blood pressure of 130/78 mm of Hg, respiratory rate of 20 breaths per min with accessory muscle use, and oxygen saturation of 94% on room air. Respiratory system examination revealed bilateral inspiratory and expiratory wheeze and bibasilar crackles, and bronchial breathing in right infraclavicular region.

**Figure 1:**
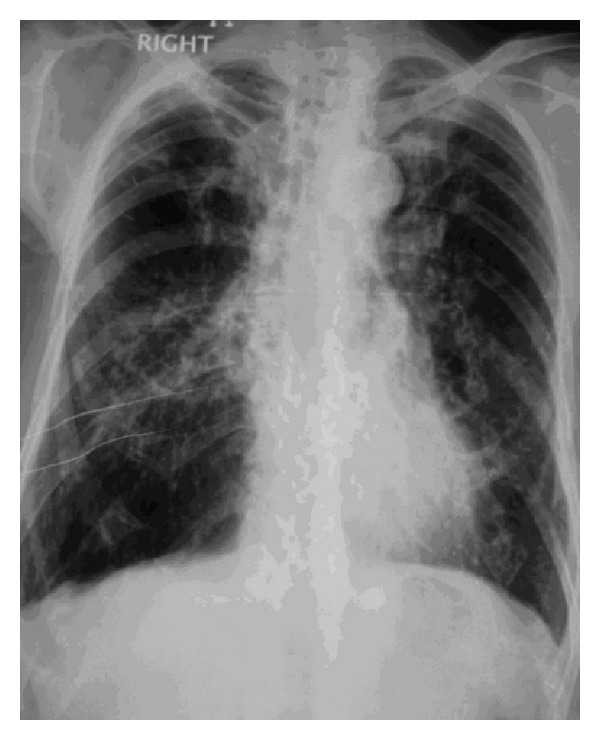


On investigation chest radiogram (Figure 1) revealed fibrotic opacities in bilateral lung fields mainly in mid and lower zones, prominent on right side with cavitary lesions in right mid and upper zones.

**Figure 2:**
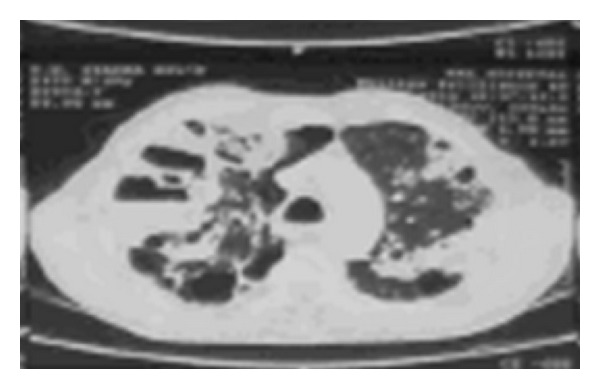


**Figure 3:**
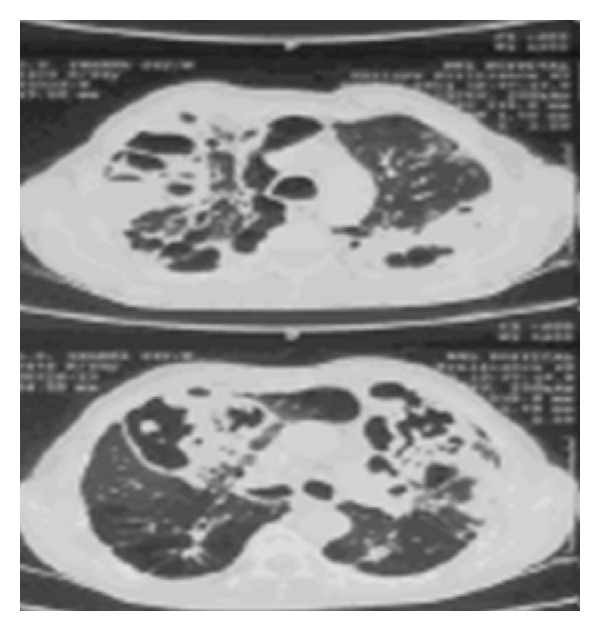


HRCT (high resolution computed tomography) of chest (Figures 2 and 3) revealed extensive emphysematous changes in both lung fields with bulla formation, central bronchiectatic changes in anterior, apical, and posterior segments of right upper lobe, apicoposterior segment of left upper lobe, lateral basal segment of right lower lobe, and apical segment of left lower lobe with peribronchial wall thickening.

We proceeded for further investigations. Ziehl-neelsen staining of sputum was negative for acid fast bacilli on several occasions and sputum for gram staining was negative for any pathogen and sputum KOH preparation failed to demonstrate any fungal hyphae. Serum immunoglobulin G specific for *Aspergillus fumigatus* was raised (192.68 iu/mL). Total serum immunoglobulin E level was 6000 iu/mL (markedly elevated). Patient was labelled as a case of ABPA and started on steroid with itraconazole. He showed initial improvement. Breathlessness and daily activity improved. Patient was shifted to insulin for better glycemic control. He was discharged and was advised followup in outpatient clinic.

**Figure 4:**
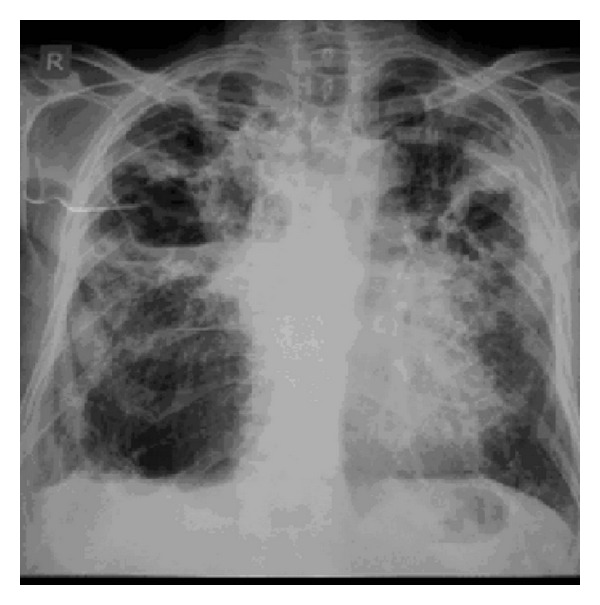


Three week after the treatment started, patient again presented to us with complaints of high grade fever with cough and mucopurulent expectoration and increased shortness of breath accompanied by pleuritic chest pain. On examination patient was restless with respiratory rate of 30 breaths/minute, blood pressure of 130/74 mm Hg, pulse rate of 96/minute, and temperature of 102°F. There was no pallor, icterus, cyanosis, clubbing, oedema, and lymphadenopathy. Oxygen saturation was 90% on room air. Respiratory examination revealed bilateral coarse crackles with expiratory wheezing with amphoric bronchial breath sounds in right infraclavicular and suprascapular areas. Steroid was withheld, itraconazole was continued, patient was stabilized with oxygen, and nebulised bronchodilators and antibiotics were added. Patient was again investigated. Investigations revealed hemoglobin of 11.7 gm%, total leukocyte count of 17000/mm^3^, peripheral smear showed toxic granules, kidney and liver function test results were within normal limits, X-ray chest (Figure 4) revealed multiple cavitary lesions with air fluid levels in right upper and mid zones and left mid zone with adjacent area of consolidation, fibrosis and volume loss. Inhomogeneous infiltrates were noted in left lower zone. Repeated Ziehl-neelsen stain of sputum were negative for AFB. Sputum for fungal culture showed insignificant fungal growth, repeated sputum for bacterial culture showed no growth. However delayed growth of Nocardia was noted on fungal plates. Sputum sample was sent for modified acid fast staining which showed the *Nocardia asteroids* (Figure 5) as weakly acid fast positive filaments. Patient was started on tablet cotrimoxazole in dose of sulphamethoxazole 2400 mg and trimethoprim 480 mg per day in divided doses. Patient became symptomatically well within a week of starting cotrimoxazole and was discharged. Patient was completely asymptomatic after one month of therapy.

## 2. Discussion

The minimum essential criteria for diagnosis of ABPA in asthma are the presence of asthma, immediate cutaneous reactivity to *Aspergillus* species or *Aspergillus fumigatus*, an elevated total serum IgE (>417 kU/l), and elevated serum IgG or IgE to *Aspergillus fumigatus*. A diagnosis of seropositive ABPA (ABPA-S) can be made with the above criteria [1]. Radiographic findings further categorize the disease. Presence of ABPA-S plus central bronchiectasis, that is, bronchiectasis in inner two-thirds of chest CT field, is necessary for a diagnosis of ABPA with central bronchiectasis (ABPA-CB). A recent article designated a third variation of this process as ABPA with central bronchiectasis and other radiologic features (ABPA-CB-ORF). These patients, in addition to satisfying criteria for ABPA-CB also had other radiologic features on CT such as pulmonary fibrosis, blebs, scarring, and fibrocavitary lesions. With progression from ABPA-S to ABPA-CB-ORF, the disease becomes increasingly severe [2].

On initial presentation, our patient fulfilled the criteria of ABPA-CB-ORF and hence we initiated the treatment for the same. The patient came back after three weeks with worsening of symptoms. Patient was reevaluated as the X-ray chest showed multiple cavitary lesions with air fluid levels and evidence of sepsis (polymorph nuclear leucocytosis with toxic granules, fever, and tachypnoea). So our patient was investigated on the line of lung abscess with reactivation of tuberculosis and invasive aspergillosis as differential diagnosis. Repeated sputum for gram stain and bacterial culture were sterile, sputum smears for Ziehl-Neelsen staining were negative for acid fast organisms, and sputum for fungal culture showed insignificant growth. We reviewed the literature for cavitatory lesions in immunodeficient individuals, and found that pneumonia is typically caused by *Staphylococcus aureus* [3] or *Pseudomonas aeruginosa* [4], and *Mycobacterium tuberculosis* [5–7] is rarely reported with* Rhodococcus equi* [8–10] and *Nocardia asteroids* [11–17].

Staphylococcal pneumonia [3] is frequently severe and typically occurs in relatively debilitated patients. Predisposing factors are age > 65 years, alcoholism, chronic bronchopulmonary disease, immunodepression, renal failure, and diabetes [18]. Pseudomonal pneumonia typically occurs in the setting of cystic fibrosis and other immunocompromised settings but its exact association in ABPA setting is still unknown. Both of them are readily cultured on Blood Agar medium. Tuberculosis is the commonest opportunistic infection in HIV infected patients; other risk factors for tuberculosis are birth in a country where tuberculosis is endemic, diabetes, presence of head and neck malignancy, haematologic malignancy, corticosteroid use, and treatment with tumor necrosis factor inhibitor [5–7]. Sputum smear with ZN staining is positive and more so in cavitatory disease. *Rhodococcus equi* is a gram-positive coccobacillus commonly isolated from soil, common cause of pneumonia in young horses. Risk factors in human are advanced human immunodeficiency virus infection (CD4 T-lymphocyte count of 200 cells/mm^3^ or less), hematologic malignancies, and use of chronic corticosteroids and other immunosuppressive agents [8–10]. No case has ever been reported in India.

Majority of patients with pulmonary nocardiosis have identifiable risk factors mainly corticosteroid therapy and other immunosuppressant therapy or some evidence of immunosuppression [12]. Nocardiosis is a life-threatening infection [17]. The association between nocardiosis and solid organ transplantation [14], HIV infection with CD4 cell count <50/cumm [15], male gender [16], steroid therapy, and immunosuppressant therapy [12] has been observed in few studies. COPD is common respiratory disease among patients with pulmonary nocardiosis [13]. COPD is commonly treated with corticosteroids. Patients have frequent infections. Bacteria colonizing the lower airways alter ciliary motility and cause epithelial damage, thereby facilitating the presence of *Nocardia* [11]. A similar phenomenon is found in patients with cystic fibrosis and bronchiectasias, who also present with epithelial changes [11]. Our patient was a longstanding asthmatic, who had developed COPD. His HRCT revealed extensive emphysematous changes in both lung with bullae formation, extensive bronchiectatic changes with peribronchial wall thickenings. He had developed ABPA-CB-ORF and had been treated with corticosteroids. He was also a diabetic, thus he had all the risk factors to develop pulmonary nocardiosis.

The clinical symptoms and CXR findings of pulmonary nocardiosis are nonspecific [19], similar to those caused by other bacteria [20, 21]. The illness tends to have a protracted course and the diagnosis is often delayed [22]. To reduce the delay in diagnosis and treatment, testing for *Nocardia* spp. should be performed in patients with risk factors for pulmonary nocardiosis and pneumonia which have not responded to treatment. Microbiological studies of respiratory tract specimens obtained by noninvasive methods are effective [23]. However, if the patient cannot expectorate, respiratory tract specimens obtained by invasive methods have a reasonable yield [23, 24]. Microscopy and culture are not difficult [22]; however, because of the slow growth of the organisms, cultures should be kept for at least 30 days [22, 24]. The microbiology laboratory should be informed that *Nocardia* spp. is suspected as the bacteria require specific media, a long culture period and modified Ziehl-nelsen staining for direct demonstration [24, 25]. In immunosuppressed patients, a positive sputum culture is more likely to indicate disease and not colonization, and the patients must be treated. Sulphonamides have been the mainstay of therapy of Nocardiosis. Trimethoprim sulphamethoxazole (TMP-SMX) combination has been usually used for treatment. There have been reports of poor treatment responses in patients of nocardiosis treated with TMP-SMX combination alone [26, 27]. Combination therapies with cotrimoxazole, amikacin, and cephalosporin or imipenem have been recommended as empirical therapy in serious, CNS, and disseminated cases [28]. Linzeolid offers an additional option. Therapy is given for a protracted period as relapses may occur [29]. There are few case reports of TMP-SMX resistance in* Nocardia* spp. from India [26] and most of the isolates even of Nocardia farcinica are susceptible to TMP-SMX [30]. Our patient had only pulmonary nocardiosis and showed a good response to treatment with TMP-SMX alone. He has been treated for 16 weeks and is doing well after finishing treatment.

## 3. Conclusion

All the patients with the risk factors like COPD, bronchiectasis, cystic fibrosis, and any other immunocompromised state, with nonresponsive pneumonia must be evaluated for rare infections like nocardia. Delay in diagnosis and treatment can be life threatening. Resistance is an emerging concern and combination therapy is an option in serious and disseminated disease.

## Figures and Tables

**Figure 5:**